# Immunogenetics of Multiple Sclerosis in Romanian Patients: Preliminary Data

**DOI:** 10.3390/ijms26157628

**Published:** 2025-08-06

**Authors:** Alexandra Elena Constantinescu, Ion Mărunțelu, Andreea Pleșa, Carmen Adella Sîrbu, Florentina Cristina Pleșa, Andreia Ioana Constantinescu, Ileana Constantinescu

**Affiliations:** 1 Faculty of Medicine, “Carol Davila” Medical University Bucharest, 020021 Bucharest, Romania; alexandra.constantinescu08@gmail.com (A.E.C.); andreea.plesa@rez.umfcd.ro (A.P.); ileana.constantinescu@imunogenetica.ro (I.C.); 2 ”Emil Palade” Center of Excellence for Young People in Scientific Research (EP-CEYR), Academy of Romanian Scientists, 030167 Bucharest, Romania; 3 Fundeni Centre for Immunogenetics and Virology, Fundeni Clinical Institute, 022328 Bucharest, Romania; 4 Neurology Department, Dr. Carol Davila Central Military Emergency University Hospital, 010825 Bucharest, Romania; carmen.sirbu@umfcd.ro (C.A.S.); cristina.plesa@umfcd.ro (F.C.P.); 5 Academy of Romanian Scientists, 030167 Bucharest, Romania; 6 Clinical Neuroscience Department, University of Medicine and Pharmacy “Carol Davila”, 020021 Bucharest, Romania

**Keywords:** HLA, genes, multiple sclerosis

## Abstract

Multiple sclerosis (MS) is a chronic autoimmune disease characterized by the immune system attacking the central nervous system, leading to demyelination and neurodegeneration. This work investigates the relationship between specific human leukocyte antigen (HLA) polymorphisms and MS, aiming to reveal the immunogenetic background of this disease for more individualized management approaches. This study employed a case–control design, analyzing HLA allele frequencies in 179 MS patients and 200 control subjects using next-generation sequencing, The key findings indicate significant associations between several HLA Class I and II alleles and MS, including HLA-B*35:03:01:03, HLA-C*04:01:01:14, HLA-DRB1*15:01:01:26, and HLA-DQA1*05:05:01:02. These findings emphasize the critical role of HLA molecules in MS Romanian patients.

## 1. Introduction

Multiple sclerosis (MS) is a chronic autoimmune disease in which the body’s immune system incorrectly turns against central nervous system (CNS) components [1]. This misdirected attack gradually erodes the myelin sheath, an insulating layer surrounding neuronal axons, leading to neurodegeneration over time [2,3,4]. MS arises from a complex interaction between genetic predisposition, environmental exposures, and infections, particularly with EBV. While no single cause explains all cases, these factors consistently contribute to immune system dysfunction and CNS damage, leading to MS development [4,5,6]. Among the multiple genetic factors explored to date, the human leukocyte antigen (HLA) system is one of the most challenging.

The HLA region, located on the short arm of chromosome 6, is one of the most genetically diverse regions in the human genome. This extensive polymorphism makes the immune system highly adaptable, allowing for the recognition of an enormous array of pathogens. However, this same variation, while enabling flexible immune defense, can also lead to errors, such as the presentation of self-derived peptides that cause T cells to attack the body’s own tissues.

HLA genes have a central role in genetic susceptibility to MS, primarily through specific alleles in the HLA Class II region, especially the HLA-DRB1 locus [4]. HLA-DRB1*15:01 contributes to MS by increasing immune gene expression through epigenetic changes, specifically DNA hypomethylation in a critical gene regulatory region [7]. Carriers of this allele show enhanced immune system activity against the nervous system, with increased expression of HLA-DRB1 and related immune genes, particularly in immune cells like monocytes [7,8,9]. This risk is amplified by environmental exposures, modulated by gender and other immunogenetic factors, making MS a complex disease [10].

The association between HLA-DRB1*15:01 and MS appears stronger in women and may vary by ethnic genetic background [8]. Other genetic aspects, such as variants in the vitamin D receptor, can also modulate the risk but to a lesser extent [8]. Elevated methylation levels near HLA-DRB1 exon 2 have been shown to suppress HLA-DRB1 expression, conferring a protective effect against MS [7]. These findings underscore the significance of HLA gene expression levels in determining disease risk [7].

Our work investigated the relationship between HLA polymorphisms and MS. We examined how the frequency of specific alleles differs between Romanian patients diagnosed with MS and the control group. By conducting this work on our MS patients, we aimed to elucidate the immunogenetic pathogenesis of MS and contribute to more individualized approaches to diagnosis, treatment, and prognosis.

## 2. Results

A total of 179 patients with multiple sclerosis (MS) and 200 healthy controls were included in this study. The demographic and clinical characteristics of the cohort are summarized in Table 1.

In the patient cohort, the predominant clinical form was relapsing-remitting MS (RRMS), representing 88.8% of cases. The mean disease duration was 12.23 years, and the median EDSS score indicated a moderate level of disability.

### 2.1. HLA Allele Associations with MS

Several alleles showed significant associations with disease in our group of patients. Among Class I alleles, HLA-B*35:03:01:03 (*p* = 0.0004, OR = 7.42) and HLA-C*04:01:01:14 (*p* = 0.0002, OR = 3.50) were significantly more frequent in MS patients compared to controls (*p_c_* < 0.05, Bonferroni-adjusted). Among Class II alleles, DRB1*15:01:01:26 (*p* < 0.00001, OR = 96.25) and DQA1*05:05:01:02 (*p* < 0.0001, OR = 2.73) showed strong associations with MS (*p_c_* < 0.05). All HLA alleles identified in the MS patient cohort, alongside their corresponding counts in the control group, are listed in Supplementary Table S1.

The statistical power for detecting each significant association was high, exceeding 95% in all cases (see Table 2).

Through gender-stratified analysis, we further investigated whether gender modifies the effect of alleles that demonstrated a significant association with MS risk in the overall analysis, unadjusted for gender. The HLA-DRB1*15:01:01:26 allele demonstrated a significant association with MS risk in both women (OR = 35.74; *p*-value < 0.0001) and men (OR = 81.80; *p*-value < 0.0001) after Bonferroni correction. Additionally, the HLA-DQA1*05:05:01:02 allele showed a significant association with MS risk only in the female subgroup (OR = 3.67; *p*-value = 0.0067) after Bonferroni correction. None of the other associations evaluated reached statistical significance in either subgroup after the Bonferroni correction was applied. Detailed results are presented in Table 3.

### 2.2. Association of HLA Alleles with SPMS Phenotype

The analysis did not reveal any statistically significant differences in allele frequencies between the MS phenotype subgroups (*p* > 0.05). The detailed frequencies for the primary associated alleles across these subgroups are presented in Table 4.

### 2.3. Association of HLA Alleles with Disease Severity (EDSS Score)

No significant associations were found between the identified HLA alleles and the age of MS onset or disease severity, as measured by the EDSS score (*p* > 0.05 for all comparisons). A summary of these clinical correlations is provided in Table 5.

## 3. Discussion

The contribution of HLA allele polymorphisms in the pathogenesis of multiple sclerosis (MS) remains a significant topic for consideration. This is due to both the immunogenetic complexity involved and the strong biological link between abnormal antigen presentation and processing via the HLA Class I or II pathway and the activation of autoimmune responses.

Moving from a general analysis of HLA allele groups to identifying specific genetic subtypes, such as HLA-DRB1*15:01:01:26 versus the broader HLA-DRB1*15:01 group, marks a substantial leap forward in medical research. It is similar to swapping a regional map for a detailed street map: we gained a much clearer understanding of what happens and precisely where it happens. When we work with broad groups of alleles, we can only make general assumptions about their role in disease. However, once a specific subtype is identified, we can formulate far more precise hypotheses about how that particular genetic variation influences biological processes at the molecular level. By pinpointing exact genetic variations, researchers can begin to decode the molecular mechanisms by which a disease develops and progresses. Certain HLA subtypes may present specific protein fragments that trigger aggressive autoimmune responses, unlike others in the same allelic group. Understanding these mechanisms enables the development of personalized therapies and targeted drugs that minimize unwanted side effects.

Our study confirms that HLA Class I and II alleles play a key role in the immunopathogenesis of MS. Using high-resolution next-generation sequencing (NGS), we identified statistically significant associations between MS and the following alleles: HLA-B*35:03:01:03, HLA-C*04:01:01:14, HLA-DRB1*15:01:01:26, and HLA-DQA1*05:05:01:02. The complete absence of the HLA-DRB1*15:01:01:26 subtype in our control group suggests that this allele is rare in the general Romanian population. However, its presence in the MS group indicates that, when identified, it confers a very high risk of disease development.

The discovery of an association between MS and the Class I subtypes HLA-B*35:03:01:03 and HLA-C*04:01:01:14 is a significant finding, as these specific high-resolution alleles have not been previously reported in connection with MS. This finding underscores the critical importance of our sequencing methodology in revealing the true polymorphic associations of these genes with MS. It also redirects some of the immunogenetic focus to the crucial role of CD8+ cytotoxic T cells. We hypothesize that subtle variations within these alleles may alter the conformation of the peptide-binding pocket. This structural change could favor the presentation of oligodendrocyte-derived self-antigens, thereby marking these myelin-producing cells for direct destruction by CD8^+^ T cells and contributing to demyelination.

For Class II alleles, such as HLA-DRB1*15:01:01:26 and HLA-DQA1*05:05:01:02, the mechanisms might be multifaceted. First, the fine structure of these molecules, revealed by NGS, could directly influence the repertoire of myelin peptides presented to CD4^+^ T cells. Unique variations in these subtypes may dictate which specific fragments of myelin proteins are displayed to the immune system. If a particular subtype is more efficient at presenting an autoreactive peptide, the risk of triggering an autoimmune response will increase. Second, nucleotide variations that define the DRB1*15:01:01:26 subtype, including non-coding subtypes, may impact processes such as splicing efficiency or mRNA stability. This could lead to the production of a greater number of HLA molecules on the cell surface, and increased density might result in a more robust and sustained activation of autoreactive T cells, thereby amplifying the immune response.

Our gender-stratified analysis provides important insights into how gender may modulate the effect of HLA alleles on the risk of multiple sclerosis (MS). We observed a significant association between the HLA-DRB1*15:01:01:26 allele and MS risk in both women and men. This suggests that the genetic influence of this allele on MS susceptibility is powerful and acts independently of sex-specific factors that might modulate overall risk or disease severity.

In contrast, the HLA-DQA1*05:05:01:02 allele showed a significant association with MS risk exclusively in the female subgroup. This result is particularly relevant as it provides a possible partial genetic explanation for the increased incidence of MS in women. It suggests that the genetic vulnerability conferred by this allele is exacerbated or predominantly manifested in a female biological context, possibly through interactions with sex-specific hormonal, immunological, or environmental factors. Although the HLA-B*35:03:01:03 and HLA-C*04:01:01:14 alleles did not reach statistical significance in any subgroup, it is important to note that this could be due to the lower statistical power of the subgroups and not necessarily due to the complete absence of an effect. Smaller sample sizes in stratified analyses may limit the ability to detect associations with more minor effects. Therefore, gender-stratified analyses in future genetic studies of multiple sclerosis are essential to identify complex interactions and better understand the distinct pathogenesis of the disease in each sex.

Building upon the established understanding of HLA’s role, our findings further refine the picture of MS pathogenesis. It is well-known that HLA Class II molecules are central to MS pathogenesis. They present myelin fragments (e.g., MBP, MOG, and PLP) to CD4+ T cells in peripheral lymphoid tissues, prompting their differentiation into inflammatory Th1 (IFN-γ) and Th17 (IL-17) subsets [11,12,13]. These activated T cells then cross the compromised blood–brain barrier (BBB) [11,12]. Within the CNS, reactivated CD4+ T cells release pro-inflammatory cytokines, while microglia and macrophages degrade myelin via enzymes and reactive oxygen species (ROS) [13,14]. Simultaneously, CD8+ cytotoxic T cells target oligodendrocytes, escalating damage [13,14]. B cells also contribute by producing myelin autoantibodies, accelerating demyelination and sustaining inflammation [15,16]. Ultimately, MS evolves into a self-sustaining autoimmune attack [17,18]. Persistent inflammation damages axons and disrupts neural signaling, leading to hallmark symptoms like muscle weakness, impaired coordination, sensory deficits, and visual disturbances [19,20,21]. The consistent identification of HLA-DRB1*15:01 across various populations, including our Romanian cohort (*p* = 0.00011 for HLA-DRB1*15:01 and *p* < 0.00001 for HLA-DRB1*15:01:01:26), highlights its strong association with MS. Our study distinctively employed NGS, which provides a more comprehensive characterization of HLA gene polymorphisms compared to some previous studies [7,22,23,24,25,26,27].

Regarding HLA-DQA1*05:05, there is limited direct evidence from other populations specifically linking this allele to MS. Most research points to other DQA1 alleles or haplotypes, such as DQA1*01:02 and DQA1*05:01, as having associations with MS susceptibility or protection [11,25]. Notably, in Japanese populations, DQA1*05:03 (distinct from DQA1*05:05) was associated with neuromyelitis optica spectrum disorder (NMOSD) but not MS [12]. Our study found a significant association for the specific high-resolution allele HLA-DQA1*05:05:01:02 with MS risk, which contrasts with the lack of association reported for the broader HLA-DQA1*05:05 allele in some other studies [28]. This contradiction illustrates a fundamental principle of MS immunogenetics: the role of an allele can be modulated or even reversed by a linkage disequilibrium with other alleles in proximity, as well as by the specific genetic background of the population. This observation underscores how high-resolution sequencing can precisely identify which specific genetic variations, even within broader allelic groups, are truly linked to disease susceptibility.

Our findings indicate that none of the identified HLA alleles significantly influence the age of MS onset or disease severity, as measured by the EDSS. This aligns with results from a study by Lisa F. Barcellos et al. involving 1336 MS families [29].

A key strength of this study is its statistical power, which validates the robustness of the identified genetic associations. Power analyses performed individually for each significant allele revealed power values ranging from 95% to 99%. These values, which far exceed the conventional threshold of 80%, demonstrate that the sample size was adequate not only for a single effect but also for the entire set of primary outcomes. Thus, we can state with a high degree of confidence that the reported associations are statistically valid, and the risk of missing other real effects (type II errors) is minimal.

Beyond this statistical validity, the analysis of the diagnostic performance of the alleles revealed a nuanced clinical profile. While their specificity as markers was very high, sensitivity overall was limited. An example is the DRB1*15:01:01:26 allele, which demonstrated a specificity of 100% and was not present in any individual in the control group, making it a powerful risk marker. Its sensitivity was only 10.61%, indicating that most MS patients do not carry this allele. This high specificity and low sensitivity are common for genetic markers in complex diseases. It underscores the conclusion that, although the identified alleles are valuable risk factors at the population level, they cannot be used as stand-alone diagnostic tests, as their absence does not exclude the disease.

However, the results should be interpreted with caution due to several limitations, which may also explain potential discrepancies with other research. Specifically, our single-center study, conducted on a limited patient cohort and employing a specific HLA gene sequencing technique, may restrict the generalizability of our findings and contribute to variations when compared to studies with different population genetics, sample sizes, or methodologies. Furthermore, our use of asymptotic tests and allele-focused analysis means that our allele-specific conclusions require careful consideration and may differ from those of studies using other analytical methods. Haplotype analysis was not performed due to limitations in statistical power. Although our sample size can identify primary allelic associations, larger cohorts are needed to minimize type II errors in polygenic diseases such as MS. Furthermore, significant demographic differences in gender and age were present between the patient and control groups. Therefore, future research priorities include investigating haplotypes in the Romanian population, employing better demographic matching, and utilizing multicenter cohorts to confirm and expand upon our findings.

From a future perspective, although the current sensitivity and specificity data do not allow a firm recommendation of these results, further research in this area is essential. As replication studies on larger cohorts validate these associations and eventually identify alleles with increased predictive value, we propose that HLA typing should be considered in the diagnostic protocol of MS patients. This could provide valuable prognostic information in the future and contribute to the development of personalized therapies.

## 4. Materials and Methods

Our work is a case–control study comparing MS patients’ immunogenetic makeup with controls. We enrolled 179 patients with MS, diagnosed according to established clinical criteria. The control group comprised 200 healthy, unrelated individuals whose samples were collected from the National Bone Marrow Donor Registry. The key matching criteria ensured all control subjects were confirmed to be in good health at the time of donation, without known autoimmune or chronic inflammatory diseases. Furthermore, they were selected to have the same distribution of geographic regions within Romania between cases and controls. This matching process was performed specifically to minimize the risk of population stratification, which is a known confounder in genetic association studies, particularly for the HLA system. All participants provided written informed consent, and the study protocol was approved by the Ethics Committee of Fundeni Clinical Institute. The study adhered to the Declaration of Helsinki principles.

All blood samples from both patients and controls were collected on Ethylenediaminetetraacetic acid (EDTA) for the extraction of DNA (QIAamp^®^ DNA Mini and Blood Mini Handbook, Hilden, Germany) and further HLA genotyping.

The identification of HLA allele polymorphisms was performed at Fundeni Clinical Institute—Centre for Immunogenetics and Virology using next-generation sequencing, Illumina platform (MiaFora, Immucor, Dreieich, Germany). The frequencies of HLA Class I (HLA-A, -B, -C) and Class II (HLA-DRB1, -DQB1, -DQA1, -DPB1) alleles were compared between MS patients and healthy controls. Allele frequencies were determined via direct counting in MS patients and controls using Microsoft Excel for Microsoft 365 MSO (Version 2506 Build 16.0.18925.20076).

Statistical differences in allele percentages between MS patients and controls were assessed via cross-tabulation in SPSS version 21.0 (IBM Corp., Armonk, NY, USA). The χ^2^ test was employed for expected values > 5, and Fisher’s exact test was used for expected values ≤ 5. Odds ratios with 95% confidence intervals (95% CIs) were calculated to quantify the strength of association for each HLA allele with MS. For the calculation of the odds ratio (OR) and 95% confidence interval (CI), in cases where one or more cells in the 2 × 2 contingency table had a value of zero, a Haldane–Anscombe continuity correction was applied (adding 0.5 to all cells in the table) [30]. This adjustment allowed the avoidance of division by zero and the calculation of finite estimates of the OR and the standard error of log (OR) required for the CI. Statistical significance was set at *p* < 0.05. To avoid the risk of identifying false-positive allelic associations; all significant probability values obtained were corrected for multiple testing using the Bonferroni correction, where the number of comparisons was equal to the total number of alleles identified in the patient group at each locus (33 for HLA-A, 99 for HLA-B, 55 for HLA-C, 84 for HLA-DRB1, 44 for HLA-DQB1, 46 for HLA-DQA1, and 51 for HLA-DPB1).

To assess the statistical power of the identified associations, we performed a post hoc power analysis for each allele that reached statistical significance. The power obtained was calculated using a two-tailed z-test with a significance level of α = 0.05. For each allele, the odds ratio (OR) observed in our study and the corresponding baseline probability of the event were entered into the calculation. The calculations were performed using the Demidenko method with variance correction [31]. In addition, to determine the discriminative capacity of each statistically significant allele, we calculated sensitivity and specificity analyses, thus evaluating their ability to differentiate between cases and controls.

## 5. Conclusions

Our study provides further evidence for the strong link between HLA gene polymorphisms and MS immunogenetic pathogenesis in Romanian MS patients. We identified the specific HLA Class I and II alleles associated with MS risk, highlighting the importance of high-resolution sequencing and gender-stratified analyses for a deeper understanding of this disease.

## Figures and Tables

**Table 1:** Demographic data and clinical characteristics.

Characteristics	Patients with MS (N = 179)	Controls (N = 200)	*p*-Value
Age (years; mean, SD)	45.94 ± 11.47	50.27 ± 20.92	<0.05
Gender (female, %)	70.4%	48.0%	<0.05
Ethnic origin (Caucasian, %)	100.0%	100.0%	
Age of disease onset (years; mean, SD)	33.70 ± 9.82	-	-
Duration of disease (years; mean, SD)	12.23 ± 7.86	-	-
Type of onset:		-	-
*Optic neuritis*	24.2%	-	-
*Motor*	40.0%	-	-
*Sensory*	33.7%	-	-
*Vestibular syndrome*	2.1%	-	-
Form of disease:			
*Primary progressive multiple sclerosis (PPMS) (%)*	4.5%	-	-
*Secondary progressive multiple sclerosis (SPMS) (%)*	6.7%	-	-
*Relapsing-remitting multiple sclerosis (RRMS) (%)*	88.8%	-	-
EDSS score (median, IQR)	3.5 (2.0–6.0)	-	-
Therapeutic status (% under DMT)	98.9% (177/179)	-	-
First-line therapy:		-	-
*Beta-interferon (all forms) (%)*	30.2%	-	-
*Teriflunomide (%)*	19.0%	-	-
*Dimethyl fumarate (%)*	7.8%	-	-
*Glatiramer acetate (%)*	5.0%	-	-
High efficiency therapy:		-	-
*Ocrelizumab (%)*	16.2%	-	-
*Natalizumab (%)*	8.9%	-	-
*Fingolimod (%)*	4.5%	-	-
*Cladribine (%)*	4.5%	-	-
*Siponimod (%)*	2.2%	-	-
*Ponesimod (%)*	0.6%	-	-

**Table 2:** Statistical and diagnostic performance parameters for significantly associated HLA alleles.

Allele (n in MS Patients/n in Controls)	Odds Ratio (OR)	95% CI	*p*-Value	*p_c_*-Value	Statistical Power (1-β)	Sensitivity	Specificity
HLA-B*35:03:01:03 (19/3)	7.42	2.18–25.28	0.0004	0.0436	0.952 (95.2%)	5.30%	99.30%
HLA-C*04:01:01:14 (35/12)	3.50	1.80–6.86	0.00021	0.0113	0.971 (97.1%)	9.80%	97.00%
HLA-DRB1*15:01:01:26 (38/0)	96.25 *	5.89–1572.81 *	<0.0001	<0.0001	0.980 (98.0%)	10.60%	100.00%
HLA-DQA1*05:05:01:02 (63/29)	2.73	1.72–4.35	<0.0001	0.0010	0.992 (99.2%)	17.60%	92.80%

The calculation of the odds ratio (OR) and other statistical parameters was based on a total of 179 patients with multiple sclerosis (MS) and 200 healthy controls. All significant *p*-values obtained were corrected for multiple testing using the Bonferroni correction, where the number of comparisons was equal to the total number of alleles identified in the patient group at each locus (99 for HLA-B, 55 for HLA-C, 84 for HLA-DRB1, and 46 for HLA-DQA1). *p_c_* = *p*-value after Bonferroni correction * Due to the absence of alleles in the controls, the OR and 95% CI were calculated using Haldane–Anscombe continuity correction.

**Table 3:** Gender-stratified analysis of the association between HLA alleles and MS risk.

Allele	Gender Group (n in MS Patients/n in Controls)	Odds Ratio (OR)	95% CI	*p*-Value	*p_c_*-Value
HLA-B*35:03:01:03	Females (14/1)	11.24	1.46–86.21	0.0082	0.6885
	Males (5/2)	5.10	0.97–26.74	0.0459	2.3395
HLA-C*04:01:01:14	Females (25/4)	5.18	1.77–15.14	0.0018	0.0912
	Males (10/8)	2.60	1.00–6.81	0.0788	-
HLA-DRB1*15:01:01:26	Females (21/0)	35.74 *	2.15–593.90 *	<0.0001	<0.0001
	Males (17/0)	81.80 *	4.87–1375.22 *	<0.0001	<0.0001
HLA-DQA1*05:05:01:02	Females (46/11)	3.67	1.85–7.31	0.0002	0.0067
	Males (17/18)	2.02	0.99–4.10	0.0757	-

The calculation of the odds ratio (OR) and other statistical parameters was based on a total of 126 female and 53 male patients with MS, as well as 96 female and 104 male healthy controls. All significant *p*-values obtained were corrected for multiple testing using the Bonferroni correction. The number of comparisons for the female patient group was equal to the number of alleles identified at each locus: 84 for HLA-B, 50 for HLA-C, 75 for HLA-DRB1, and 40 for HLA-DQA1. For the male patient group, the numbers were 51 for HLA-B, 34 for HLA-C, 45 for HLA-DRB1, and 31 for HLA-DQA1. *p_c_* = *p*-value after Bonferroni correction. * Due to the absence of alleles in the controls, the OR and 95% CI were calculated using Haldane–Anscombe continuity correction.

**Table 4:** Frequency of HLA alleles in patients with different phenotypes of multiple sclerosis.

Allele	Allele Frequency in SPMS (%) (n/Total)	Allele Frequency in RRMS (%) (n/Total)	Allele Frequency in PPMS (%) (n/Total)	*p*-Value (SPMS vs. RRMS)	*p*-Value (SPMS vs. PPMS)
HLA-B*35:03:01:03	4.2% (1/24)	5.0% (16/318)	12.5% (2/16)	>0.05	>0.05
HLA-C*04:01:01:14	4.2% (1/24)	9.8% (31/318)	18.8% (3/16)	>0.05	>0.05
DRB1*15:01:01:26	20.8% (5/24)	9.4% (30/318)	18.8% (3/16)	>0.05	>0.05
DQA1*05:05:01:02	29.2% (7/24)	17.3% (55/318)	6.3% (1/16)	>0.05	>0.05

**Table 5:** Correlation between the presence of HLA alleles and clinical parameters.

Allele	Average EDSS in Allele Carriers ± SD	Average EDSS in Allele Non-Carriers ± SD	*p*-Values (EDSS)	Mean Age of Onset in Allele Carriers ± SD	Mean Age of Onset in Allele Non-Carriers ± SD	*p*-Values (Age of Onset)
HLA-B*35:03:01:03	2.63 ± 2.11	2.59 ± 1.88	>0.05	35.68 ± 10.17	33.60 ± 9.80	>0.05
HLA-C*04:01:01:14	2.39 ± 1.85	2.62 ± 1.90	>0.05	36.03 ± 12.00	33.46 ± 9.54	>0.05
DRB1*15:01:01:26	2.99 ± 2.01	2.55 ± 1.88	>0.05	34.95 ±9.61	33.56 ± 9.84	>0.05
DQA1*05:05:01:02	2.47 ± 1.92	2.62 ± 1.89	>0.05	33.10 ± 9.09	33.84 ± 9.70	>0.05

## Data Availability

The dataset is available on request from the authors.

## Supplementary Materials

The following supporting information can be downloaded at: https://www.mdpi.com/article/10.3390/ijms26157628/s1.
