# Supplementary material for: Immunogenetics of Multiple Sclerosis in Romanian Patients: Preliminary Data
Source: Int J Mol Sci. 2025 Aug 6;26(15):7628. doi: 10.3390/ijms26157628 (PMC12347847; doi:10.3390/ijms26157628)
Supplement: Supplementary file 1 [file ijms-26-07628-s001.zip › ijms-3687614-supplementary.pdf]

Table S1. HLA alleles identified in the MS patient cohort and their corresponding counts in the control group.

HLA allele	PPMS		RRMS		SPMS		Total MS patients (PPMS+RRMS+SPMS)		Control	
	n	(%)	n	(%)	n	(%)	n	(%)	n	(%)
A*01:01:01:01	3	0.8	41	11.5	3	0.8	47	13.1	51	12.8
A*01:01:01:03	0	0.0	2	0.6	0	0.0	2	0.6	1	0.3
A*02:01:01:01	2	0.6	73	20.4	5	1.4	80	22.3	110	27.5
A*02:01:01:05	0	0.0	3	0.8	0	0.0	3	0.8	1	0.3
A*02:01:01:08	0	0.0	2	0.6	0	0.0	2	0.6	0	0.0
A*02:01:01:09	0	0.0	1	0.3	0	0.0	1	0.3	0	0.0
A*02:05:01:01	0	0.0	2	0.6	1	0.3	3	0.8	2	0.5
A*02:08:01	0	0.0	1	0.3	0	0.0	1	0.3	0	0.0
A*02:17:02:01	0	0.0	1	0.3	0	0.0	1	0.3	1	0.3
A*03:01:01:01	0	0.0	30	8.4	3	0.8	33	9.2	29	7.3
A*03:02:01:01	0	0.0	1	0.3	0	0.0	1	0.3	1	0.3
A*11:01:01:01	0	0.0	25	7.0	1	0.3	26	7.3	28	7.0
A*23:01:01:01	1	0.3	9	2.5	2	0.6	12	3.4	10	2.5
A*24:02:01:01	2	0.6	29	8.1	3	0.8	34	9.5	43	10.8
A*24:02:01:04	0	0.0	2	0.6	0	0.0	2	0.6	3	0.8
A*24:02:01:05	0	0.0	3	0.8	0	0.0	3	0.8	3	0.8
A*24:03:01:01	0	0.0	3	0.8	0	0.0	3	0.8	0	0.0
A*25:01:01:01	1	0.3	7	2.0	0	0.0	8	2.2	14	3.5
A*26:01:01:01	2	0.6	24	6.7	0	0.0	26	7.3	19	4.8
A*29:02:01:01	0	0.0	4	1.1	0	0.0	4	1.1	3	0.8
A*30:01:01:01	1	0.3	4	1.1	1	0.3	6	1.7	6	1.5
A*30:02:01:01	0	0.0	7	2.0	0	0.0	7	2.0	0	0.0
A*31:01:02:01	0	0.0	3	0.8	1	0.3	4	1.1	9	2.3
A*31:01:02:04	0	0.0	1	0.3	0	0.0	1	0.3	0	0.0
A*32:01:01:01	3	0.8	19	5.3	2	0.6	24	6.7	14	3.5
A*32:01:01:02	0	0.0	0	0.0	1	0.3	1	0.3	0	0.0
A*33:03:01:01	0	0.0	4	1.1	1	0.3	5	1.4	4	1.0
A*66:01:01:01	0	0.0	4	1.1	0	0.0	4	1.1	1	0.3
A*68:01:01:02	1	0.3	7	2.0	0	0.0	8	2.2	8	2.0
A*68:01:02:01	0	0.0	1	0.3	0	0.0	1	0.3	2	0.5
A*68:01:02:02	0	0.0	3	0.8	0	0.0	3	0.8	6	1.5
A*68:02:01:01	0	0.0	1	0.3	0	0.0	1	0.3	1	0.3
A*74:03:01:01	0	0.0	1	0.3	0	0.0	1	0.3	0	0.0
B*07:02:01:01	1	0.3	10	2.8	2	0.6	13	3.6	22	5.5
B*07:02:01:11	0	0.0	1	0.3	0	0.0	1	0.3	0	0.0
B*08:01:01:01	1	0.3	18	5.0	2	0.6	21	5.9	30	7.5
B*08:01:01:02	0	0.0	1	0.3	0	0.0	1	0.3	1	0.3
B*08:01:01:15	0	0.0	1	0.3	0	0.0	1	0.3	0	0.0
B*08:01:01:37	0	0.0	1	0.3	0	0.0	1	0.3	0	0.0

B*13:02:01:01	2	0.6	11	3.1	1	0.3	14	3.9	10	2.5
B*13:02:01:12	0	0.0	0	0.0	1	0.3	1	0.3	0	0.0
B*14:01:01:01	0	0.0	1	0.3	0	0.0	1	0.3	1	0.3
B*14:02:01:01	0	0.0	4	1.1	0	0.0	4	1.1	18	4.5
B*15:01:01:01	0	0.0	6	1.7	0	0.0	6	1.7	16	4.0
B*15:01:01:04	0	0.0	3	0.8	0	0.0	3	0.8	1	0.3
B*15:01:01:06	0	0.0	2	0.6	0	0.0	2	0.6	0	0.0
B*15:01:01:45	0	0.0	1	0.3	0	0.0	1	0.3	0	0.0
B*15:17:01:01	0	0.0	1	0.3	0	0.0	1	0.3	0	0.0
B*15:18:01:02	0	0.0	1	0.3	0	0.0	1	0.3	1	0.3
B*18:01:01:01	0	0.0	1	0.3	0	0.0	1	0.3	1	0.3
B*18:01:01:02	0	0.0	4	1.1	0	0.0	4	1.1	10	2.5
B*18:01:01:06	0	0.0	1	0.3	0	0.0	1	0.3	0	0.0
B*18:01:01:10	1	0.3	0	0.0	0	0.0	1	0.3	1	0.3
B*18:01:01:11	0	0.0	1	0.3	0	0.0	1	0.3	0	0.0
B*18:01:01:12	0	0.0	2	0.6	0	0.0	2	0.6	0	0.0
B*18:01:01:16	0	0.0	1	0.3	0	0.0	1	0.3	2	0.5
B*18:01:01:22	1	0.3	2	0.6	0	0.0	3	0.8	0	0.0
B*18:01:01:26	0	0.0	2	0.6	0	0.0	2	0.6	6	1.5
B*18:01:01:28	0	0.0	2	0.6	0	0.0	2	0.6	1	0.3
B*18:01:01:32	0	0.0	1	0.3	0	0.0	1	0.3	0	0.0
B*18:01:01:33	0	0.0	2	0.6	0	0.0	2	0.6	1	0.3
B*18:01:01:45	0	0.0	1	0.3	1	0.3	2	0.6	0	0.0
B*18:01:01:48	0	0.0	1	0.3	0	0.0	1	0.3	0	0.0
B*18:01:01:52	0	0.0	4	1.1	0	0.0	4	1.1	20	5.0
B*18:01:01:69	0	0.0	3	0.8	0	0.0	3	0.8	0	0.0
B*18:01:01:74	0	0.0	1	0.3	0	0.0	1	0.3	0	0.0
B*18:01:01:76	0	0.0	2	0.6	0	0.0	2	0.6	0	0.0
B*18:05:01:02	0	0.0	6	1.7	0	0.0	6	1.7	0	0.0
B*27:02:01:01	0	0.0	2	0.6	0	0.0	2	0.6	2	0.5
B*27:02:01:04	0	0.0	1	0.3	0	0.0	1	0.3	3	0.8
B*27:05:02:01	0	0.0	3	0.8	0	0.0	3	0.8	1	0.3
B*27:05:02:05	1	0.3	3	0.8	0	0.0	4	1.1	2	0.5
B*35:01:01:05	0	0.0	15	4.2	1	0.3	16	4.5	27	6.8
B*35:01:01:25	0	0.0	2	0.6	0	0.0	2	0.6	0	0.0
B*35:02:01:02	0	0.0	5	1.4	0	0.0	5	1.4	1	0.3
B*35:03:01:01	0	0.0	11	3.1	0	0.0	11	3.1	6	1.5
B*35:03:01:03	2	0.6	16	4.5	1	0.3	19	5.3	3	0.8
B*35:03:01:09	0	0.0	1	0.3	0	0.0	1	0.3	0	0.0
B*35:03:01:15	0	0.0	1	0.3	0	0.0	1	0.3	0	0.0
B*35:08:01:01	0	0.0	4	1.1	0	0.0	4	1.1	10	2.5
B*37:01:01:01	0	0.0	5	1.4	0	0.0	5	1.4	5	1.3
B*38:01:01:01	1	0.3	12	3.4	1	0.3	14	3.9	15	3.8
B*39:01:01:01	0	0.0	1	0.3	0	0.0	1	0.3	0	0.0
B*39:01:01:03	0	0.0	1	0.3	0	0.0	1	0.3	8	2.0

B*39:01:01:09	0	0.0	2	0.6	0	0.0	2	0.6	0	0.0
B*39:01:01:10	0	0.0	1	0.3	0	0.0	1	0.3	0	0.0
B*39:01:01:11	0	0.0	2	0.6	0	0.0	2	0.6	0	0.0
B*39:01:01:18	0	0.0	3	0.8	0	0.0	3	0.8	0	0.0
B*39:06:02:01	0	0.0	1	0.3	0	0.0	1	0.3	3	0.8
B*40:01:02:01	0	0.0	1	0.3	0	0.0	1	0.3	6	1.5
B*40:02:01:01	0	0.0	5	1.4	0	0.0	5	1.4	3	0.8
B*40:02:01:08	0	0.0	1	0.3	0	0.0	1	0.3	1	0.3
B*40:02:01:18	1	0.3	2	0.6	1	0.3	4	1.1	1	0.3
B*40:02:01:41	0	0.0	1	0.3	0	0.0	1	0.3	0	0.0
B*40:06:01:13	0	0.0	1	0.3	0	0.0	1	0.3	0	0.0
B*40:06:01:17	0	0.0	1	0.3	0	0.0	1	0.3	0	0.0
B*40:11:01:01	0	0.0	1	0.3	0	0.0	1	0.3	0	0.0
B*41:01:01:01	0	0.0	3	0.8	0	0.0	3	0.8	4	1.0
B*41:02:01:01	1	0.3	2	0.6	1	0.3	4	1.1	0	0.0
B*44:02:01:01	0	0.0	15	4.2	0	0.0	15	4.2	18	4.5
B*44:02:01:03	0	0.0	1	0.3	0	0.0	1	0.3	0	0.0
B*44:03:01:01	0	0.0	3	0.8	0	0.0	3	0.8	4	1.0
B*44:03:01:19	1	0.3	5	1.4	0	0.0	6	1.7	7	1.8
B*44:03:01:29	1	0.3	0	0.0	0	0.0	1	0.3	0	0.0
B*44:03:02:01	0	0.0	1	0.3	0	0.0	1	0.3	0	0.0
B*44:27:01:01	0	0.0	3	0.8	0	0.0	3	0.8	3	0.8
B*45:01:01:01	0	0.0	0	0.0	1	0.3	1	0.3	0	0.0
B*45:01:01:03	0	0.0	1	0.3	0	0.0	1	0.3	0	0.0
B*47:01:01:03	0	0.0	6	1.7	0	0.0	6	1.7	0	0.0
B*49:01:01:01	0	0.0	6	1.7	2	0.6	8	2.2	13	3.3
B*49:01:01:04	0	0.0	0	0.0	1	0.3	1	0.3	0	0.0
B*50:01:01:01	0	0.0	7	2.0	1	0.3	8	2.2	2	0.5
B*51:01:01:01	0	0.0	9	2.5	1	0.3	10	2.8	6	1.5
B*51:01:01:03	0	0.0	4	1.1	1	0.3	5	1.4	4	1.0
B*51:01:01:04	1	0.3	10	2.8	2	0.6	13	3.6	14	3.5
B*51:01:01:09	0	0.0	1	0.3	0	0.0	1	0.3	0	0.0
B*51:01:01:105	0	0.0	1	0.3	0	0.0	1	0.3	0	0.0
B*51:01:01:35	0	0.0	2	0.6	0	0.0	2	0.6	0	0.0
B*51:01:01:45	0	0.0	2	0.6	0	0.0	2	0.6	5	1.3
B*51:01:01:59	0	0.0	2	0.6	0	0.0	2	0.6	1	0.3
B*51:01:01:65	0	0.0	2	0.6	0	0.0	2	0.6	4	1.0
B*51:01:01:78	0	0.0	1	0.3	0	0.0	1	0.3	0	0.0
B*51:01:01:84	0	0.0	0	0.0	1	0.3	1	0.3	0	0.0
B*52:01:01:01	0	0.0	3	0.8	0	0.0	3	0.8	1	0.3
B*52:01:01:02	0	0.0	5	1.4	0	0.0	5	1.4	6	1.5
B*53:01:01:01	0	0.0	1	0.3	0	0.0	1	0.3	2	0.5
B*55:01:01:01	1	0.3	8	2.2	0	0.0	9	2.5	9	2.3
B*56:01:01:15	0	0.0	1	0.3	0	0.0	1	0.3	0	0.0
B*57:01:01:01	0	0.0	4	1.1	0	0.0	4	1.1	8	2.0

B*58:01:01:01	0	0.0	2	0.6	1	0.3	3	0.8	5	1.3
B*58:01:01:17	0	0.0	2	0.6	1	0.3	3	0.8	0	0.0
B*58:01:01:20	0	0.0	1	0.3	0	0.0	1	0.3	0	0.0
C*01:02:01:01	0	0.0	13	3.6	1	0.3	14	3.9	16	4.0
C*02:02:02:01	1	0.3	10	2.8	0	0.0	11	3.1	20	5.0
C*02:02:02:03	0	0.0	4	1.1	1	0.3	5	1.4	2	0.5
C*02:02:02:08	1	0.3	0	0.0	0	0.0	1	0.3	0	0.0
C*02:02:02:79	0	0.0	3	0.8	0	0.0	3	0.8	0	0.0
C*02:02:02:83	0	0.0	1	0.3	0	0.0	1	0.3	0	0.0
C*03:02:02:05	0	0.0	3	0.8	1	0.3	4	1.1	0	0.0
C*03:03:01:01	1	0.3	7	2.0	0	0.0	8	2.2	19	4.8
C*03:03:01:37	0	0.0	1	0.3	0	0.0	1	0.3	0	0.0
C*03:04:01:01	0	0.0	6	1.7	0	0.0	6	1.7	13	3.3
C*03:04:01:02	0	0.0	1	0.3	0	0.0	1	0.3	2	0.5
C*04:01:01:05	0	0.0	2	0.6	0	0.0	2	0.6	2	0.5
C*04:01:01:06	0	0.0	12	3.4	1	0.3	13	3.6	14	3.5
C*04:01:01:08	0	0.0	1	0.3	0	0.0	1	0.3	1	0.3
C*04:01:01:100	0	0.0	1	0.3	0	0.0	1	0.3	0	0.0
C*04:01:01:11	0	0.0	2	0.6	0	0.0	2	0.6	2	0.5
C*04:01:01:14	3	0.8	31	8.7	1	0.3	35	9.8	12	3.0
C*04:01:01:75	1	0.3	4	1.1	0	0.0	5	1.4	1	0.3
C*04:01:01:79	0	0.0	7	2.0	0	0.0	7	2.0	0	0.0
C*04:01:01:91	0	0.0	1	0.3	0	0.0	1	0.3	0	0.0
C*04:01:102	0	0.0	1	0.3	0	0.0	1	0.3	0	0.0
C*05:01:01:01	0	0.0	1	0.3	0	0.0	1	0.3	2	0.5
C*05:01:01:02	1	0.3	13	3.6	0	0.0	14	3.9	11	2.8
C*06:02:01:01	2	0.6	27	7.5	3	0.8	32	8.9	19	4.8
C*06:02:01:02	0	0.0	5	1.4	1	0.3	6	1.7	3	0.8
C*06:02:01:03	0	0.0	0	0.0	1	0.3	1	0.3	0	0.0
C*06:02:01:97	0	0.0	1	0.3	0	0.0	1	0.3	0	0.0
C*07:01:01:01	1	0.3	17	4.7	2	0.6	20	5.6	26	6.5
C*07:01:01:109	0	0.0	1	0.3	0	0.0	1	0.3	0	0.0
C*07:01:01:131	0	0.0	1	0.3	0	0.0	1	0.3	0	0.0
C*07:01:01:16	2	0.6	21	5.9	3	0.8	26	7.3	47	11.8
C*07:01:02:01	0	0.0	1	0.3	0	0.0	1	0.3	0	0.0
C*07:02:01:01	0	0.0	9	2.5	1	0.3	10	2.8	10	2.5
C*07:02:01:03	1	0.3	6	1.7	1	0.3	8	2.2	20	5.0
C*07:02:01:136	0	0.0	0	0.0	1	0.3	1	0.3	0	0.0
C*07:04:01:01	0	0.0	3	0.8	0	0.0	3	0.8	3	0.8
C*07:04:01:03	0	0.0	2	0.6	0	0.0	2	0.6	1	0.3
C*07:06:01:01	0	0.0	1	0.3	0	0.0	1	0.3	0	0.0
C*07:18:01:01	0	0.0	2	0.6	1	0.3	3	0.8	3	0.8
C*08:02:01:01	0	0.0	4	1.1	0	0.0	4	1.1	16	4.0
C*08:02:01:02	0	0.0	1	0.3	0	0.0	1	0.3	2	0.5
C*12:02:02:01	0	0.0	8	2.2	0	0.0	8	2.2	7	1.8

C*12:03:01:01	1	0.3	39	10.9	1	0.3	41	11.5	46	11.5
C*12:03:01:02	0	0.0	1	0.3	0	0.0	1	0.3	0	0.0
C*12:03:01:13	0	0.0	4	1.1	0	0.0	4	1.1	0	0.0
C*14:02:01:01	0	0.0	8	2.2	1	0.3	9	2.5	8	2.0
C*15:02:01:01	0	0.0	12	3.4	2	0.6	14	3.9	19	4.8
C*15:04:01:01	0	0.0	2	0.6	0	0.0	2	0.6	0	0.0
C*15:05:02:01	0	0.0	1	0.3	0	0.0	1	0.3	0	0.0
C*16:01:01:01	0	0.0	4	1.1	0	0.0	4	1.1	3	0.8
C*16:02:01:01	0	0.0	4	1.1	0	0.0	4	1.1	4	1.0
C*16:04:01:01	0	0.0	3	0.8	0	0.0	3	0.8	4	1.0
C*17:01:01:05	0	0.0	2	0.6	0	0.0	2	0.6	3	0.8
C*17:01:01:09	0	0.0	1	0.3	0	0.0	1	0.3	0	0.0
C*17:03:01:01	1	0.3	2	0.6	1	0.3	4	1.1	0	0.0
DRB1*01:01:01:01	0	0.0	17	4.7	0	0.0	17	4.7	38	9.5
DRB1*01:01:01:04	0	0.0	1	0.3	0	0.0	1	0.3	0	0.0
DRB1*01:02:01:01	0	0.0	1	0.3	0	0.0	1	0.3	7	1.8
DRB1*01:02:01:02	0	0.0	1	0.3	0	0.0	1	0.3	0	0.0
DRB1*03:01:01:01	0	0.0	14	3.9	2	0.6	16	4.5	33	8.3
DRB1*03:01:01:02	0	0.0	7	2.0	0	0.0	7	2.0	1	0.3
DRB1*03:01:01:03	0	0.0	4	1.1	0	0.0	4	1.1	5	1.3
DRB1*03:01:01:08	0	0.0	1	0.3	0	0.0	1	0.3	0	0.0
DRB1*03:01:01:13	0	0.0	3	0.8	0	0.0	3	0.8	0	0.0
DRB1*03:01:01:15	0	0.0	3	0.8	0	0.0	3	0.8	0	0.0
DRB1*03:01:01:16	0	0.0	2	0.6	0	0.0	2	0.6	0	0.0
DRB1*03:01:08	0	0.0	2	0.6	0	0.0	2	0.6	0	0.0
DRB1*03:07:01:01	0	0.0	1	0.3	0	0.0	1	0.3	0	0.0
DRB1*03:07:01:02	0	0.0	1	0.3	0	0.0	1	0.3	0	0.0
DRB1*04:01:01:01	0	0.0	3	0.8	0	0.0	3	0.8	8	2.0
DRB1*04:01:01:11	0	0.0	1	0.3	0	0.0	1	0.3	0	0.0
DRB1*04:01:01:18	0	0.0	1	0.3	0	0.0	1	0.3	0	0.0
DRB1*04:02:01:01	1	0.3	9	2.5	1	0.3	11	3.1	5	1.3
DRB1*04:03:01:01	1	0.3	4	1.1	0	0.0	5	1.4	1	0.3
DRB1*04:04:01:05	1	0.3	0	0.0	0	0.0	1	0.3	6	1.5
DRB1*04:04:01:14	0	0.0	9	2.5	0	0.0	9	2.5	0	0.0
DRB1*04:05:01:04	0	0.0	2	0.6	0	0.0	2	0.6	5	1.3
DRB1*04:05:01:05	0	0.0	1	0.3	0	0.0	1	0.3	0	0.0
DRB1*04:07:01:01	0	0.0	1	0.3	0	0.0	1	0.3	3	0.8
DRB1*07:01:01:02	0	0.0	5	1.4	0	0.0	5	1.4	5	1.3
DRB1*07:01:01:04	1	0.3	3	0.8	0	0.0	4	1.1	30	7.5
DRB1*07:01:01:16	0	0.0	3	0.8	0	0.0	3	0.8	0	0.0
DRB1*07:01:01:25	0	0.0	1	0.3	0	0.0	1	0.3	0	0.0
DRB1*07:01:01:26	1	0.3	9	2.5	1	0.3	11	3.1	0	0.0
DRB1*08:01:01:01	0	0.0	9	2.5	0	0.0	9	2.5	3	0.8
DRB1*08:03:02:03	0	0.0	1	0.3	0	0.0	1	0.3	0	0.0
DRB1*08:04:01:01	0	0.0	0	0.0	1	0.3	1	0.3	0	0.0

DRB1*08:04:01:03	0	0.0	1	0.3	0	0.0	1	0.3	0	0.0
DRB1*10:01:01:03	0	0.0	3	0.8	1	0.3	4	1.1	10	2.5
DRB1*11:01:01:01	0	0.0	7	2.0	0	0.0	7	2.0	3	0.8
DRB1*11:01:01:10	0	0.0	11	3.1	1	0.3	12	3.4	0	0.0
DRB1*11:01:01:16	0	0.0	2	0.6	2	0.6	4	1.1	0	0.0
DRB1*11:01:01:19	0	0.0	2	0.6	0	0.0	2	0.6	0	0.0
DRB1*11:01:02:03	0	0.0	1	0.3	0	0.0	1	0.3	0	0.0
DRB1*11:02:01:01	0	0.0	0	0.0	1	0.3	1	0.3	0	0.0
DRB1*11:03:01	0	0.0	2	0.6	0	0.0	2	0.6	7	1.8
DRB1*11:04:01:01	1	0.3	19	5.3	0	0.0	20	5.6	47	11.8
DRB1*11:04:01:02	0	0.0	1	0.3	0	0.0	1	0.3	0	0.0
DRB1*11:04:01:03	0	0.0	11	3.1	0	0.0	11	3.1	0	0.0
DRB1*11:04:01:06	1	0.3	1	0.3	0	0.0	2	0.6	0	0.0
DRB1*11:04:01:09	1	0.3	7	2.0	1	0.3	9	2.5	0	0.0
DRB1*11:11:01	0	0.0	1	0.3	0	0.0	1	0.3	0	0.0
DRB1*11:12:01	0	0.0	1	0.3	0	0.0	1	0.3	0	0.0
DRB1*12:01:01:01	0	0.0	1	0.3	0	0.0	1	0.3	0	0.0
DRB1*12:01:01:05	0	0.0	1	0.3	0	0.0	1	0.3	2	0.5
DRB1*13:01:01:01	0	0.0	3	0.8	0	0.0	3	0.8	13	3.3
DRB1*13:01:01:03	0	0.0	1	0.3	0	0.0	1	0.3	0	0.0
DRB1*13:01:01:10	0	0.0	2	0.6	0	0.0	2	0.6	1	0.3
DRB1*13:01:01:18	0	0.0	2	0.6	0	0.0	2	0.6	0	0.0
DRB1*13:01:01:19	0	0.0	1	0.3	0	0.0	1	0.3	0	0.0
DRB1*13:01:01:20	2	0.6	7	2.0	0	0.0	9	2.5	0	0.0
DRB1*13:01:01:23	0	0.0	2	0.6	0	0.0	2	0.6	0	0.0
DRB1*13:02:01:01	0	0.0	2	0.6	0	0.0	2	0.6	1	0.3
DRB1*13:02:01:02	0	0.0	3	0.8	0	0.0	3	0.8	3	0.8
DRB1*13:02:01:03	0	0.0	0	0.0	1	0.3	1	0.3	2	0.5
DRB1*13:02:01:04	0	0.0	1	0.3	1	0.3	2	0.6	0	0.0
DRB1*13:02:01:05	0	0.0	1	0.3	0	0.0	1	0.3	0	0.0
DRB1*13:02:01:07	0	0.0	1	0.3	0	0.0	1	0.3	2	0.5
DRB1*13:02:01:11	0	0.0	1	0.3	0	0.0	1	0.3	0	0.0
DRB1*13:03:01:01	1	0.3	3	0.8	2	0.6	6	1.7	5	1.3
DRB1*13:03:01:03	0	0.0	1	0.3	0	0.0	1	0.3	0	0.0
DRB1*13:03:01:05	0	0.0	2	0.6	0	0.0	2	0.6	0	0.0
DRB1*13:05:01:01	0	0.0	0	0.0	1	0.3	1	0.3	0	0.0
DRB1*13:27:01	0	0.0	1	0.3	0	0.0	1	0.3	0	0.0
DRB1*14:01:01:01	0	0.0	2	0.6	0	0.0	2	0.6	0	0.0
DRB1*14:04:01:01	0	0.0	1	0.3	0	0.0	1	0.3	1	0.3
DRB1*14:54:01:06	0	0.0	1	0.3	0	0.0	1	0.3	0	0.0
DRB1*14:54:01:08	1	0.3	8	2.2	1	0.3	10	2.8	14	3.5
DRB1*15:01:01:01	0	0.0	4	1.1	0	0.0	4	1.1	0	0.0
DRB1*15:01:01:05	0	0.0	2	0.6	0	0.0	2	0.6	7	1.8
DRB1*15:01:01:23	0	0.0	1	0.3	0	0.0	1	0.3	0	0.0
DRB1*15:01:01:26	3	0.8	30	8.4	5	1.4	38	10.6	0	0.0

DRB1*15:02:01:03	0	0.0	4	1.1	0	0.0	4	1.1	7	1.8
DRB1*15:02:02:03	0	0.0	1	0.3	0	0.0	1	0.3	0	0.0
DRB1*16:01:01:01	0	0.0	24	6.7	0	0.0	24	6.7	25	6.3
DRB1*16:01:01:02	0	0.0	9	2.5	0	0.0	9	2.5	12	3.0
DRB1*16:02:01:02	0	0.0	4	1.1	1	0.3	5	1.4	2	0.5
DRB1*16:02:01:04	1	0.3	2	0.6	0	0.0	3	0.8	1	0.3
DQB1*02:01:01:01	0	0.0	31	8.7	2	0.6	33	9.2	39	9.8
DQB1*02:01:01:02	0	0.0	3	0.8	0	0.0	3	0.8	0	0.0
DQB1*02:01:01:07	0	0.0	2	0.6	0	0.0	2	0.6	0	0.0
DQB1*02:01:01:12	0	0.0	1	0.3	0	0.0	1	0.3	0	0.0
DQB1*02:02:01:01	2	0.6	12	3.4	2	0.6	16	4.5	29	7.3
DQB1*02:02:01:02	0	0.0	1	0.3	0	0.0	1	0.3	2	0.5
DQB1*02:02:01:06	0	0.0	2	0.6	0	0.0	2	0.6	0	0.0
DQB1*03:01:01:01	0	0.0	2	0.6	0	0.0	2	0.6	8	2.0
DQB1*03:01:01:02	1	0.3	9	2.5	0	0.0	10	2.8	9	2.3
DQB1*03:01:01:03	1	0.3	39	10.9	7	2.0	47	13.1	54	13.5
DQB1*03:01:01:04	0	0.0	2	0.6	0	0.0	2	0.6	0	0.0
DQB1*03:01:01:05	0	0.0	2	0.6	0	0.0	2	0.6	9	2.3
DQB1*03:01:01:09	2	0.6	14	3.9	0	0.0	16	4.5	18	4.5
DQB1*03:01:01:17	0	0.0	2	0.6	0	0.0	2	0.6	0	0.0
DQB1*03:01:01:18	0	0.0	3	0.8	0	0.0	3	0.8	1	0.3
DQB1*03:01:01:33	0	0.0	1	0.3	0	0.0	1	0.3	0	0.0
DQB1*03:02:01:01	1	0.3	18	5.0	1	0.3	20	5.6	25	6.3
DQB1*03:02:01:02	1	0.3	8	2.2	0	0.0	9	2.5	2	0.5
DQB1*03:02:01:04	0	0.0	1	0.3	0	0.0	1	0.3	0	0.0
DQB1*03:02:01:10	0	0.0	3	0.8	0	0.0	3	0.8	0	0.0
DQB1*03:03:02:01	0	0.0	5	1.4	0	0.0	5	1.4	9	2.3
DQB1*03:05:01	1	0.3	0	0.0	0	0.0	1	0.3	1	0.3
DQB1*03:231	0	0.0	1	0.3	0	0.0	1	0.3	0	0.0
DQB1*04:02:01:04	0	0.0	5	1.4	0	0.0	5	1.4	4	1.0
DQB1*04:02:01:08	0	0.0	1	0.3	1	0.3	2	0.6	0	0.0
DQB1*05:01:01:01	0	0.0	3	0.8	0	0.0	3	0.8	7	1.8
DQB1*05:01:01:03	0	0.0	16	4.5	0	0.0	16	4.5	35	8.8
DQB1*05:01:01:05	0	0.0	3	0.8	1	0.3	4	1.1	11	2.8
DQB1*05:02:01:01	1	0.3	35	9.8	2	0.6	38	10.6	43	10.8
DQB1*05:02:01:02	0	0.0	1	0.3	0	0.0	1	0.3	0	0.0
DQB1*05:02:01:03	0	0.0	2	0.6	0	0.0	2	0.6	0	0.0
DQB1*05:02:01:04	0	0.0	7	2.0	0	0.0	7	2.0	4	1.0
DQB1*05:02:01:08	0	0.0	1	0.3	0	0.0	1	0.3	0	0.0
DQB1*05:03:01:01	1	0.3	13	3.6	1	0.3	15	4.2	20	5.0
DQB1*05:04	0	0.0	1	0.3	0	0.0	1	0.3	3	0.8
DQB1*06:01:01:01	0	0.0	5	1.4	0	0.0	5	1.4	4	1.0
DQB1*06:02:01:01	0	0.0	19	5.3	4	1.1	23	6.4	16	4.0
DQB1*06:02:01:06	0	0.0	1	0.3	0	0.0	1	0.3	0	0.0
DQB1*06:02:01:21	2	0.6	0	0.0	0	0.0	2	0.6	0	0.0

DQB1*06:03:01:01	3	0.8	32	8.9	0	0.0	35	9.8	22	5.5
DQB1*06:03:01:03	0	0.0	1	0.3	0	0.0	1	0.3	0	0.0
DQB1*06:03:01:05	0	0.0	1	0.3	1	0.3	2	0.6	2	0.5
DQB1*06:04:01:01	0	0.0	6	1.7	1	0.3	7	2.0	8	2.0
DQB1*06:09:01:01	0	0.0	3	0.8	1	0.3	4	1.1	2	0.5
DQA1*01:01:01:01	0	0.0	1	0.3	0	0.0	1	0.3	2	0.5
DQA1*01:01:01:05	0	0.0	16	4.5	0	0.0	16	4.5	33	8.3
DQA1*01:01:02:01	0	0.0	2	0.6	0	0.0	2	0.6	5	1.3
DQA1*01:02:01:01	1	0.3	7	2.0	3	0.8	11	3.1	3	0.8
DQA1*01:02:01:02	0	0.0	1	0.3	0	0.0	1	0.3	0	0.0
DQA1*01:02:01:03	1	0.3	23	6.4	2	0.6	26	7.3	19	4.8
DQA1*01:02:01:04	0	0.0	2	0.6	1	0.3	3	0.8	8	2.0
DQA1*01:02:01:05	1	0.3	5	1.4	1	0.3	7	2.0	2	0.5
DQA1*01:02:01:11	0	0.0	2	0.6	0	0.0	2	0.6	0	0.0
DQA1*01:02:01:20	0	0.0	1	0.3	0	0.0	1	0.3	0	0.0
DQA1*01:02:01:23	0	0.0	4	1.1	1	0.3	5	1.4	0	0.0
DQA1*01:02:01:26	0	0.0	2	0.6	0	0.0	2	0.6	0	0.0
DQA1*01:02:01:28	0	0.0	1	0.3	0	0.0	1	0.3	0	0.0
DQA1*01:02:02:01	0	0.0	19	5.3	0	0.0	19	5.3	38	9.5
DQA1*01:02:02:02	0	0.0	7	2.0	1	0.3	8	2.2	0	0.0
DQA1*01:02:02:04	0	0.0	2	0.6	0	0.0	2	0.6	0	0.0
DQA1*01:02:02:05	1	0.3	15	4.2	0	0.0	16	4.5	10	2.5
DQA1*01:03:01:01	0	0.0	5	1.4	0	0.0	5	1.4	4	1.0
DQA1*01:03:01:02	2	0.6	20	5.6	0	0.0	22	6.1	23	5.8
DQA1*01:04:01:01	1	0.3	7	2.0	1	0.3	9	2.5	2	0.5
DQA1*01:04:01:03	0	0.0	1	0.3	0	0.0	1	0.3	1	0.3
DQA1*01:04:01:04	0	0.0	1	0.3	0	0.0	1	0.3	0	0.0
DQA1*01:04:01:05	0	0.0	1	0.3	0	0.0	1	0.3	0	0.0
DQA1*01:04:02:02	0	0.0	1	0.3	0	0.0	1	0.3	3	0.8
DQA1*01:04:05	0	0.0	1	0.3	0	0.0	1	0.3	0	0.0
DQA1*01:05:01:01	0	0.0	3	0.8	1	0.3	4	1.1	11	2.8
DQA1*01:06	0	0.0	1	0.3	0	0.0	1	0.3	0	0.0
DQA1*02:01:01:01	2	0.6	19	5.3	1	0.3	22	6.1	36	9.0
DQA1*03:01:01:01	3	0.8	29	8.1	1	0.3	33	9.2	26	6.5
DQA1*03:03:01:01	0	0.0	2	0.6	0	0.0	2	0.6	12	3.0
DQA1*03:03:01:05	0	0.0	2	0.6	0	0.0	2	0.6	2	0.5
DQA1*04:01:01:03	0	0.0	4	1.1	0	0.0	4	1.1	4	1.0
DQA1*04:01:02:01	0	0.0	1	0.3	1	0.3	2	0.6	0	0.0
DQA1*04:02	0	0.0	1	0.3	0	0.0	1	0.3	0	0.0
DQA1*05:01:01:01	0	0.0	6	1.7	0	0.0	6	1.7	2	0.5
DQA1*05:01:01:02	0	0.0	17	4.7	2	0.6	19	5.3	31	7.8
DQA1*05:01:01:03	0	0.0	7	2.0	0	0.0	7	2.0	7	1.8
DQA1*05:01:01:06	0	0.0	6	1.7	0	0.0	6	1.7	0	0.0
DQA1*05:05:01:02	1	0.3	55	15.4	7	2.0	63	17.6	29	7.3
DQA1*05:05:01:05	0	0.0	1	0.3	0	0.0	1	0.3	1	0.3

DQA1*05:05:01:20	0	0.0	2	0.6	0	0.0	2	0.6	12	3.0
DQA1*05:05:01:28	0	0.0	2	0.6	0	0.0	2	0.6	0	0.0
DQA1*05:05:01:30	2	0.6	0	0.0	0	0.0	2	0.6	0	0.0
DQA1*05:05:01:32	1	0.3	9	2.5	0	0.0	10	2.8	0	0.0
DQA1*05:09:01:01	0	0.0	2	0.6	1	0.3	3	0.8	1	0.3
DQA1*06:01:01:03	0	0.0	2	0.6	0	0.0	2	0.6	0	0.0
DPB1*01:01:01:01	1	0.3	13	3.6	1	0.3	15	4.2	17	4.3
DPB1*01:01:01:03	1	0.3	0	0.0	0	0.0	1	0.3	0	0.0
DPB1*02:01:02:01	0	0.0	3	0.8	0	0.0	3	0.8	0	0.0
DPB1*02:01:02:05	0	0.0	29	8.1	2	0.6	31	8.7	74	18.5
DPB1*02:01:02:13	0	0.0	4	1.1	0	0.0	4	1.1	0	0.0
DPB1*02:01:02:17	0	0.0	2	0.6	0	0.0	2	0.6	4	1.0
DPB1*02:01:02:29	0	0.0	2	0.6	0	0.0	2	0.6	1	0.3
DPB1*02:01:02:32	0	0.0	1	0.3	0	0.0	1	0.3	1	0.3
DPB1*02:01:02:77	0	0.0	16	4.5	1	0.3	17	4.7	0	0.0
DPB1*03:01:01:01	2	0.6	10	2.8	4	1.1	16	4.5	26	6.5
DPB1*03:01:01:04	0	0.0	4	1.1	0	0.0	4	1.1	0	0.0
DPB1*03:01:01:09	1	0.3	1	0.3	0	0.0	2	0.6	1	0.3
DPB1*03:01:01:10	0	0.0	1	0.3	0	0.0	1	0.3	0	0.0
DPB1*03:01:01:19	1	0.3	2	0.6	0	0.0	3	0.8	0	0.0
DPB1*04:01:01:01	0	0.0	7	2.0	0	0.0	7	2.0	1	0.3
DPB1*04:01:01:05	1	0.3	22	6.1	1	0.3	24	6.7	37	9.3
DPB1*04:01:01:06	1	0.3	42	11.7	4	1.1	47	13.1	89	22.3
DPB1*04:01:01:10	1	0.3	2	0.6	0	0.0	3	0.8	0	0.0
DPB1*04:01:01:110	0	0.0	20	5.6	2	0.6	22	6.1	0	0.0
DPB1*04:01:01:28	0	0.0	1	0.3	0	0.0	1	0.3	0	0.0
DPB1*04:02:01:01	0	0.0	15	4.2	1	0.3	16	4.5	8	2.0
DPB1*04:02:01:02	0	0.0	25	7.0	1	0.3	26	7.3	38	9.5
DPB1*04:02:01:04	0	0.0	1	0.3	0	0.0	1	0.3	1	0.3
DPB1*04:02:01:06	0	0.0	5	1.4	0	0.0	5	1.4	10	2.5
DPB1*04:02:01:08	0	0.0	7	2.0	0	0.0	7	2.0	2	0.5
DPB1*04:02:01:09	0	0.0	7	2.0	0	0.0	7	2.0	2	0.5
DPB1*04:02:01:16	0	0.0	5	1.4	0	0.0	5	1.4	0	0.0
DPB1*05:01:01:01	1	0.3	5	1.4	0	0.0	6	1.7	4	1.0
DPB1*05:01:01:03	1	0.3	1	0.3	0	0.0	2	0.6	1	0.3
DPB1*06:01:01:01	0	0.0	2	0.6	0	0.0	2	0.6	4	1.0
DPB1*09:01:01:01	0	0.0	1	0.3	0	0.0	1	0.3	0	0.0
DPB1*10:01:01:01	2	0.6	12	3.4	0	0.0	14	3.9	12	3.0
DPB1*10:01:01:02	0	0.0	1	0.3	0	0.0	1	0.3	0	0.0
DPB1*10:01:01:03	0	0.0	1	0.3	0	0.0	1	0.3	0	0.0
DPB1*104:01:01:01	0	0.0	6	1.7	1	0.3	7	2.0	7	1.8
DPB1*104:01:01:03	0	0.0	2	0.6	1	0.3	3	0.8	2	0.5
DPB1*105:01:01:01	0	0.0	2	0.6	0	0.0	2	0.6	0	0.0
DPB1*11:01:01:01	0	0.0	3	0.8	0	0.0	3	0.8	7	1.8
DPB1*13:01:01:01	0	0.0	3	0.8	0	0.0	3	0.8	4	1.0

DPB1*13:01:01:02	0	0.0	5	1.4	0	0.0	5	1.4	6	1.5
DPB1*14:01:01:01	1	0.3	9	2.5	1	0.3	11	3.1	6	1.5
DPB1*14:01:01:02	0	0.0	1	0.3	0	0.0	1	0.3	0	0.0
DPB1*15:01:01:01	0	0.0	1	0.3	2	0.6	3	0.8	3	0.8
DPB1*15:01:01:02	0	0.0	2	0.6	0	0.0	2	0.6	0	0.0
DPB1*17:01:01:01	1	0.3	7	2.0	0	0.0	8	2.2	6	1.5
DPB1*17:01:01:02	0	0.0	3	0.8	0	0.0	3	0.8	1	0.3
DPB1*18:01:01:01	0	0.0	1	0.3	1	0.3	2	0.6	0	0.0
DPB1*19:01:01:01	1	0.3	0	0.0	0	0.0	1	0.3	1	0.3
DPB1*23:01:01:01	0	0.0	0	0.0	1	0.3	1	0.3	11	2.8
DPB1*23:01:01:02	0	0.0	1	0.3	0	0.0	1	0.3	0	0.0
DPB1*88:01	0	0.0	2	0.6	0	0.0	2	0.6	0	0.0
